# Combating yellow fever virus with 7-deaza-7-fluoro-2′-C-methyladenosine

**DOI:** 10.1128/aac.01889-24

**Published:** 2025-04-14

**Authors:** Julia C. LeCher, Vivian Vasconcelos Costa, Lauren N. Rust, Leda C. Bassit, Dharmeshkumar Patel, Sahar Rezaei, Justin Moua, Felipe Rocha da Silva Santos, Matheus Rodrigues Goncalves, Celso Martins Queroz-Junior, Fernanda Martins Marim, Longhu Zhou, Sujin Lee, Tamara McBrayer, Ramyani De, Niloufar Azadi, Mohammad Salman, Keivan Zandi, Franck Amblard, Benjamin Burwitz, Mauro M. Teixeira, Raymond F. Schinazi

**Affiliations:** 1 Center for ViroScience and Cure, Laboratory of Biochemical Pharmacology, Department of Pediatrics, Emory University School of Medicine and Children’s Healthcare of Atlanta 12239, Atlanta, Georgia, USA; 2 Department of Morphology, Instituto de Ciências Biológicas, Universidade Federal de Minas Gerais 113014 , Belo Horizonte, State of Minas Gerais, Brazil; 3 Division of Pathobiology & Immunology, Oregon National Primate Research Center, Oregon Health & Science University 467005, Portland, Oregon, USA; 4 Department of Biochemistry and Immunology, Instituto de Ciências Biológicas, Universidade Federal de Minas Gerais 113014 , Belo Horizonte, Minas Gerais, Brazil; IrsiCaixa Institut de Recerca de la Sida, Barcelona, Spain

**Keywords:** yellow fever virus, nucleoside analogs, antiviral agents, flavivirus, DFA, 2-FBU

## Abstract

Yellow fever virus (YFV) is a deadly zoonotic flavivirus endemic in tropical/sub-tropical Africa and South America transmitted by mosquito vector (*Aedes aegypti*; *Haemagogus leucocelaenus*) to humans and non-human primates. There are no approved antiviral agents for YFV. We previously identified 7-deaza-7-fluoro-2′-C-methyladenosine (DFA) with anti-YFV activity. Interestingly, DFA exhibits pan-activity in vitro against flaviviruses, such as dengue, Japanese encephalitis, Zika, and hepatitis C. This study aimed to expand DFA’s anti-flavivirus profile. DFA exhibited potent sub-micromolar anti-YFV activity *in vitro* against both the vaccine strain (YFV-17D) and a viscerotropic clinical YFV isolate (DakH1279) concomitantly with low cellular cytotoxicity and no notable mitochondrial toxicity. *In vivo*, efficacy was assessed against both YFV-17DD and a human clinical isolate in A129 and AG129 mouse flavivirus infection models, respectively. DFA significantly reduced virus replication in the livers of YFV-infected mice and the hallmarks of YFV-induced liver damage, including alanine transaminase levels and indocyanine green clearance. Collectively, this work identifies DFA as a potent YFV inhibitor and lays the groundwork for further therapeutic development as a YFV and, potentially, pan-flavivirus therapeutic.

## INTRODUCTION

Yellow fever virus (YFV) is a potentially lethal zoonotic flavivirus transmitted to humans and non-human primates (NHPs) by mosquitos found in tropical and sub-tropical regions of Africa and South America (1–3). A persistent sylvatic transmission cycle between NHPs and mosquitos retains the virus in circulation and confounds attempts at eradication. Acute infection is rapid, lasting 3–6 days, and often misdiagnosed. If control over acute infection fails, inflammation persists, and patients may evolve to severe disease manifestation characterized by virus dissemination to vital organs (commonly the liver, kidneys, spleen, and brain), leading to hemorrhaging with a ~50% mortality rate (1–5).

Mortality rates vary across outbreaks and depend on both the strain of YFV and the locale of the outbreak. Strategies to limit and/or prevent YFV epidemics include enhanced mosquito control, rapid screening, wide-scale vaccination programs, and the development of antiviral therapeutics. Despite significant effort, YFV remains a persistent threat, with ~200,000 cases reported annually (2). Deadly epidemics have occurred throughout history in the Americas, Africa, and Europe. A recent epidemic in South America (2016–2019) saw high levels of severe infection and mortality attributed to rapid dissemination of YFV in a relatively unvaccinated population. Furthermore, testing in vaccinated individuals revealed drops in anti-YFV titers over time, challenging the one-time vaccination strategy (6, 7). This epidemic highlighted the need for anti-YFV therapeutics, of which there are none.

**Fig 1:**
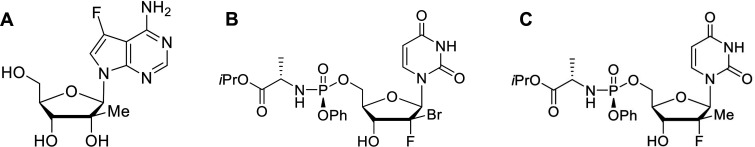
Chemical structures of compounds studied herein: **A**) DFA, **B**) 2-FBU, and **C**) sofosbuvir.

Recently, strides have been made to repurpose existing or generate new anti-YFV therapeutics (2). Using a focused nucleoside library screening effort, our team identified several nucleoside analogs as potential YFV inhibitors (8). Sofosbuvir (Fig. 1C), FDA-approved for treatment of hepatitis C virus (HCV), was shown by us and others to be active against YFV *in vitro* (8–11), though it had little efficacy when administered post-infection in a murine model (9). Of note, we identified two other potential anti-YFV nucleoside analogs: i) a 2′-fluoro,2′-bromouridine phosphoramidate prodrug (2-FBU; Fig. 1B) with potent anti-YFV 17D (vaccine strain) activity *in vitro* (8, 10) and in a murine model (10) with low cytotoxicity (CC_50_ = 68.9–>100 µM [8, 10], cell line-dependent); and ii) a 7-deaza-7-fluoro-2′-C-methyladenosine (DFA; Fig. 1A) previously shown to inhibit not only YFV (8) but other vector-borne flaviviruses, including dengue virus (DENV; EC_50_ = 0.5–1.0 µM, serotype-dependent [12]), Japanese encephalitis virus (JEV; EC_50_ = 0.9 µM [12]), and Zika virus (ZIKV) (EC_50_ = 3.7 µM [13]), as well as the non-vector borne HCV (EC_50_ = 0.07–0.9 µM [14]) with low cytotoxicity (CC_50_ = >100–>200 µM [12–14], cell line-dependent) and mitochondrial toxicity (MTNC = >50 µM [12]) *in vitro* and a favorable pharmacokinetic profile (14). In this work, we expanded on DFA’s efficacy as an anti-YFV agent *in vitro* and in a murine model. DFA exhibited potent sub-micromolar activity against both YFV-17D (EC_50_ = 0.9–5.5 µM) and a highly lethal, viscerotropic strain YFV-DakH1279 (EC_50_ = 2.4 µM) *in vitro*. In murine models, DFA provided projection against both YFV-17DD and a clinical YFV strain by significantly reducing YFV-mediated liver dysfunction and viral replication.

## MATERIALS AND METHODS

### Cells, media, and virus

Cells used in this study are as follows: i) a human hepatocarcinoma liver epithelial cell line (Huh-7; JCRB0403) purchased from JCRB Cell Bank (JCRB, Japan); ii) an African green monkey kidney cell line (Vero; American Type Culture Collection [ATCC] CCL-81) purchased from ATCC (Manassas, VA); iii) primary human peripheral blood mononuclear (PBM) cells (isolated in-house from pooled three-donor healthy human blood (NY Blood Bank, NY, NY) as previously described [10]); and iv) monocyte-derived macrophages (MDMs) generated from PBM cells isolated via Ficoll–Hypaque density gradient centrifugation, CD14^+^-positive selection (autoMACS; Miltenyi Biotech, Germany), and 8 days of treatment with granulocyte–macrophage colony-stimulating factor (GM-CSF) (Miltenyi Biotech, Germany) as previously described (10, 15). Media compositions: (1) Huh-7–Dulbecco’s modified eagle medium (DMEM), 10% fetal bovine serum (FBS), 100 U/mL penicillin–streptomycin (pen–strep), and 2 µM L-glutamine (L-glut) for YFV-17D experiments or 10% DMEM/F12 supplemented with 1% Cytiva HyClone antibiotic/antimycotic solution, 1% L-glut, 1% sodium pyruvate for YFV-Dak1279 experiments (2); Vero–DMEM, 10% FBS, 100 U/mL pen–strep, and 2 µM L-glut (3, 4), PBM and MDM–RPMI 1640 medium, 10% FBS, 100 IU/mL IL-2, and 100 U/100 µg/mL pen/strep. For all experiments, cells were grown at 37°C in a 95% O_2_, 5% CO_2_ incubator. The live attenuated YFV-17D strain was obtained from the Center for Disease Control and Prevention and propagated in-house in Huh-7 cells as previously described (10) to generate working virus stocks with a titer of 10^6^ TCID_50_/mL. The macaque-adapted clinical isolate YFV-DakH1279 was derived as previously described (16). To generate working YFV-DakH1279 stocks, Vero cells were plated in a T225 flask (10^7^ cells) in media. After 24 h, cells were infected with high titer YFV-DakH1279 (TCID_50_ = 10^6^) at a multiplicity of infection (MOI) of 0.1 in 9 mL of media. Cells were incubated at 37°C in a 95% O_2_, 5% CO_2_ incubator for 2 h. After incubation, 21 mL of media was added, and cells were incubated at 37°C in a 95% O_2_, 5% CO_2_ incubator for 7 days. Supernatants were collected at days 4 and 7 post-infection (p.i.) and centrifuged at 830 ×*g* for 4 min. The supernatant was aliquoted and titered as previously described (16).

### Synthesis of compounds

Nucleoside analogs (DFA, 2-FBU, and sofosbuvir) were synthesized in-house using published protocols (17–20) and determined to be ≥98% pure by liquid chromatography–mass spectrometry and nuclear magnetic resonance spectroscopy. Compound structures are shown in Fig. 1.

### MTS cytotoxicity assay

Cytotoxicity was evaluated as previously described (8) in PBM, Vero, Huh-7, and MDM cells for their ability to process 3-(4,5-dimethylthiazol-2-yl)-5-(3-carboxymethoxyphenyl)-2-(4-sulfophenyl)-2H-tetrazolium (MTS) (21). Specifically, 10^3^ (Vero, Huh-7) or 10^5^ (MDM, PBM) cells/well were plated in 96-well plates using respective media and treated with 1, 10, or 100 µM compound for 3 (Vero), 4 (Huh-7 and MDM), or 5 (PBM) days. Control groups were treated with 0.1, 1, and 10 µM of cycloheximide (CHX) or left untreated. MTS reagent (Promega, Madison, WI, USA) was added (15 µL for Huh-7 and Vero cells; 20 µL for MDM and PBM cells), and optical density (OD) was measured at 490 nm on a BioTek Epoch microplate plate reader (Agilent, Santa Clara, USA). Background absorbance was subtracted, and CC_50_ was determined using the Chou and Talalay method (22). Experiments were run in triplicate three times (*n* = 9).

### Mitochondrial toxicity assay

Mitochondrial toxicity was assayed as previously described with slight modification (23). Huh-7 cells were grown in DMEM supplemented with 10% dialyzed FBS and seeded at 10^3^ cells/well. After 24 h, cells were treated with 1, 10, or 50 µM compound. Negative control groups were untreated cells and cells treated with 10 µM of lamivudine (3TC) (Microbiologica Quimicae Farmaceutica Ltda, Rio de Janeiro, Brazil). Positive control groups were treated with 10 µM CHX and 1, 10, or 50 µM of zalcitabine (ddC; Baker Cummins Pharmaceuticals, Sanford, ME, USA). Dosing was performed every 3 days to simulate the dosage employed for antiviral screening. On day 14, cells were lysed, and RNA/DNA was copurified via an RNeasy 96 Extraction Kit (Qiagen, Hilden, Germany) following the manufacturer’s protocol. Downstream qPCR was multiplexed to detect mitochondrial (mt) cytochrome c oxidase subunit 2 (COXII) (mtDNA; sense primer, 5′-TGCCCGCCATCATCCTA-3′; probe, 5′-tetrachloro-6-carboxyfluorescein-TCCTCATCGCCCTCCCATCCC-TAMRA-3′; and antisense probe, 5′-CGTCTGTTATGTAAAGGATGCGT-3′) and ribosomal RNA (endogenous rRNA control; sense primer, 5′-GCGCGGCTACAGCTTCA-3′; probe, 5′−6-FAM-CACCACGGCCGAGCGGGA-TAMRA-3′; and antisense probe, 5′-TCTCCTTAATGTCACGCACGAT-3′) (23) using PerfeCTa qPCR ToughMix (Quantabio, Beverly, MA, USA) Master Mix on Lightcycler 480 II (Roche, Germany) according to the manufacturer’s protocol. Data were normalized to rRNA and IC_50_ determined by variable slope four-parameter model linear regression on GraphPad Prism v10 (GraphPad Software, Inc., San Diego, CA) and reported as a ratio of mtDNA/nDNA. Experiments were performed in duplicate three times (*n* = 6).

### *In vitro* anti-YFV-17D assessment of compounds via RT-PCR

Anti-YFV-17D activity of compounds was determined by qRT-PCR as previously described (8, 10). For two-dimensional (2D) Huh-7 cultures, cells were plated and grown to confluency overnight (10^5^ cells/well). For MDM cultures, cells were used 8 days post-differentiation. For three-dimensional (3D) spheroids, cultures were used 1 week post-initial culture. For all cultures, cells were treated with twofold serial dilutions of compound (0–10 µM) in respective base media supplemented with 2% FBS for 1 h, then infected with YFV-17D at an MOI of 0.01 (Huh-7) or 0.1 (MDM, spheroids) for 3 days in the presence of compound. After 3 days, Huh-7 and MDMs were collected in RLT buffer (Qiagen, Hilden, Germany). 3D spheroids were collected through a high-throughput technique we previously described (10) that utilizes successive wash and pellet steps to remove Matrigel and lyse cells in RLT buffer. RNA was extracted using RNeasy 96 Extraction Kit (Qiagen, Hilden, Germany), and viral load was determined by qRT-PCR on Lightcycler 480 (Roche, Germany) using a 6-carboxyfluorescein (FAM)-labeled probe with primers against YFV as previously described (8, 10) (forward primer, 5′-AGA AGA TTG GTT AGA TGA TGA TAG T-3′; reverse primer, 5′-TTC CAT CTC TAA TTG AGG TTG AAC C-3′; and probe, 5′-TC CTC ACT GCC GTC TTG TTG ACC-3′) (IDT, Newark, New Jersey, USA). All 2D experiments were run in triplicate three times (*n* = 9), while 3D spheroid experiments were run in duplicate three times (*n* = 6). Ct values were quantified against a standard curve and averaged, and effective concentrations reducing viral yield by 50% (EC_50_) and 90% (EC_90_) were calculated by variable slope four-parameter model linear regression on GraphPad Prism v10 (GraphPad Software, Inc., San Diego, CA). Experiments were run in triplicate three times (*n* = 9).

### *In vitro* anti-YFV-17D and anti-YFV-DakH1279 assessment of compounds via foci-forming assay

All antiviral evaluations via foci-forming assays were performed in Huh-7 cells grown to ~90% confluency (10^5^) in a 96-well plate treated with twofold serial dilutions of compound (0–10 µM). For YFV-17D ELIspots, cells were infected with YFV-17D [260 focus forming units (FFU)/well] for 2 h. After 2 h, the virus was removed, and the cells were treated with compound in 1% methyl cellulose for 3 days. For YFV-DaKH1279 ELIspots, infections were carried out in the BSL3 facility at Oregon National Primate Research Center, Portland, OR. Cells were infected with YFV-DakH1279 (500 FFU/well). After 2 h, the virus was removed, and cells were treated with indicated compound dilutions in media. After 2 days, infected cell media were transferred to uninfected Huh-7 cells plated in a 96-well plate at ~90% confluency. Virus was incubated with cells for 2 h, then removed, and cells were overlayed with 1% methylcellulose for 48 h. For all experiments, methylcellulose was aspirated, and cells were fixed in 4% paraformaldehyde at room temperature for 20 min. For YFV-17D ELIspots, fixed cells were blocked (Pierce Clear Milk Blocking Buffer; Thermo Fisher, USA), incubated overnight with a pan-flavivirus group antigen primary antibody (anti-flavivirus C1:D1-4G2-4-15 Mouse IgG 2a; Absolute Antibody, Centennial, Colorado), then treated with secondary goat anti-mouse HRP antibody (Invitrogen) for 1 h, followed by detection of viral antigen with KPL TrueBlue Peroxidase Substrate (SeraCare, Milford, MA, USA) and imaging at 4× on a CTL plate reader. For YFV-DakH1279 ELIspots, cells were incubated with primary antibodies bt-3A8 (kind gift from Dr. Slifka [16]) and 2D12 (Millipore, Sigma) in 0.1% saponin permeabilization buffer containing 2% FBS, followed by a secondary antibody (goat α-ms IgG-HRP; Invitrogen) each for 1 h at room temperature with washing (saponin buffer) in between. The HRP secondary was detected with 1-Step Ultra TMB-Blotting Solution (Thermo Scientific). Foci were visualized and numerated on an ImmunoSpot CTL reader, and antiviral activity was quantified against a standard curve. EC_50_ and EC_90_ values were calculated by variable slope four-parameter model linear regression on GraphPad Prism v10 (GraphPad Software, Inc., San Diego, CA). Experiments were run in duplicate three times (*n* = 6).

### Molecular modeling

Predictive modeling of DFA binding to the active site of the YFV RdRp (NS5) was performed utilizing the Schrödinger suite as previously described (10). In brief, the published crystal structure of NS5 (PDB ID: 6QSN) (24) was used, and ADP was transferred from the highly homologous HCV RdRp (PDB ID: 4WTD) (25) to YFV RdRp. From ADP, DFA-DP was generated using the build module of the Schrodinger suite. The generated structures were minimized, and the Prime MM-GBSA dG value was calculated using the Prime MM-GBSA module of Schrödinger.

### *In vivo* anti-YFV activity in A129 and AG129 mice

#### A129/AG129 mice and *in vivo* YFV infections

*In vivo* experiments were performed as previously described with slight modification (10). Male and female type I interferon receptor-deficient mice (A129) aged 8–10 weeks or type I and II interferon receptor-deficient mice (AG129) on SV129/Ev background aged 8–9 weeks were used. All mice were kept under specific pathogen-free conditions at 23°C on a 12 h light/12 h dark cycle with food and water provided *ad libitum* at the Immunopharmacology Laboratory at ICB-UFMG, Brazil. A129 mice were infected with YFV-17DD (10^6^ PFU) in 100 µL PBS by intravenous (i.v.) route in the tail vein, and experiments were conducted at the NB2 facility at the Immunopharmacology Lab. AG129 mice were infected with 10^4^ PFU in 200 µL PBS of a clinical YFV isolate (WT306 [26]) by i.v. route, and experiments were conducted in a BSL3 laboratory at ICB/UFMG. For all infections, a group of infected mice was treated with 10 mg/kg of DFA at 1 hpi and every 24 h thereafter by intraperitoneal (i.p.) route. A mock vehicle group received virus plus an equal volume of saline. In A129-YFV-17DD infections, an additional uninfected DFA-only group was evaluated. Clinical signs and body weight loss were evaluated daily. On day 3 (A129 mice) or 5 pi (AG129 mice), mice were euthanized to obtain blood, spleen, and liver. Body mass loss percentage and clinical signs of disease were monitored daily. All surgeries were performed under ketamine/xylazine anesthesia and under approval of the local ethical committee (access #84/2018 and #337/2023).

#### Hematological analysis

Blood samples were collected from the vena cava vein of mice using heparin-containing syringes. The total and differential leukocytes were determined using Nihon Kohden’s Celltac MEK-6500K Hemocytometer.

#### Indocyanine green assay

Indocyanine green (ICG, Sigma-Aldrich) was administered i.v. (20 mg/kg). After 20 min, blood samples were collected, centrifuged at 2,000 *g* for 10 min, and serum was used to quantify systemic ICG levels via spectrophotometry (800 nm).

#### Alanine aminotransferase

To determine ALT levels, blood samples were centrifuged, and the serum was collected to measure ALT levels using a kinetic kit (Bioclin, Brazil) following the manufacturer’s instructions.

#### RT-qPCR

For YFV quantification in mouse liver samples, RT-qPCR was performed. RNA was isolated using a QIAamp Viral RNA Mini Kit (Qiagen, Hilden, Germany). Total RNA was adjusted to 50 ng/3 µL and submitted to one-step RT-qPCR using the Quantinova Probe RT-PCR Kit (Qiagen, Hilden, Germany) and the 7500 Fast Real-time PCR (Applied Biosystem, Waltham, MA). Primers: forward: 5´-GCTAATTGAGGTGYATTGGTCTGC-3´; reverse: 5´-CTGCTAATCGCTCAAMGAACG-3´; and probe: 5′-ATCGAGTTGCTAGGCAATAAA CAC-3′.

#### Viral titration

YFV titers in mouse liver and plasma were measured by plaque assay using Vero cells, as previously described (26). The plaque counts were converted to PFU per g of tissue.

#### Histopathological liver analysis

Liver samples were stained with hematoxylin and eosin (H&E), and the histopathology analyses were evaluated according to criteria of cellular infiltration, hepatocyte swelling, and degeneration, and scored on a four-point scale (0, absent; 1, slight; 2, moderate; 3, marked; and 4, severe) in each analysis. Two sections for each animal were examined, and results were plotted as the mean of damage values in each mouse. Image acquisition and analysis were performed using an Olympus BX Microscope (Olympus).

#### Statistical analysis

Between groups, means were compared by Student’s *t-*test; among groups, means were analyzed by one-way analysis of variance (ANOVA). Data were plotted using GraphPad Prism v8 (GraphPad Software Inc., San Diego, CA).

## RESULTS

### Toxicity and anti-YFV-17D activity of compounds

DFA presents with low cytotoxicity and mitochondrial toxicity in different cell lines, including Vero, Huh-7, human skeletal muscle (RD), baby hamster kidney cells, and neural stem cells (8, 12–14). To expand upon DFA’s toxicity profile, we assayed toxicity in both PBM and MDM cells since YFV is a blood-borne pathogen, and macrophages serve as a primary viral reservoir, as well as a means to transport virus to target organs (Table 1) (4, 27). Vero and Huh-7 cells were also evaluated to validate previous toxicity findings. DFA exhibited low cytotoxicity and mitochondrial toxicity, similar to previous reports (Table 1). DFA had potent anti-YFV-17D activity in human hepatomas (Huh-7: EC_50_ = 0.9 ± 0.6 µM, EC_90_ = 3.4 ± 0.5 µM; Table 1; Fig. S1), primary human macrophages (MDM: EC_50_ = 0.5 ± 0.4 µM, EC_90_ = 1.0 ± 0.6 µM; Table 1), and 3D hepatoma spheroids (EC_50_ = 5.5 ± 1.3 µM, EC_90_ = 12.6 ± 1.9 µM; Table 1). Effective concentrations for 2-FBU and sofosbuvir were similar to those previously published (8–10). The purine nucleoside DFA had similar anti-YFV activity in culture as the pyrimidine nucleosides 2-FBU and sofosbuvir.

**TABLE 1:** Anti-YFV-17D activity and toxicity of compounds

Compound	Anti-YFV-17D activity^*a*^						Cytotoxicity^*b*^ CC_50_ (µM)^*d*^				Mitotoxicity^*c*^ (Huh-7)
	Huh-7^*f*^		MDM^*f*^		3D spheroids^*f*^						
	EC_50_ (µM)^*e*^	EC_90_ (µM)^*e*^	EC_50_ (µM)^*e*^	EC_90_ (µM)^*e*^	EC_50_ (µM)^*e*^	EC_90_ (µM)^*e*^	PBM^*f*^	Vero^*f*^	Huh-7^*f*^	MDM^*f*^	IC_50_ (µM) MtDNA/ nDNA^*g*^
DFA	0.9 ± 0.6	3.4 ± 0.5	0.5 ± 0.4	1.0 ± 0.6	5.5 ± 1.3	12.6 ± 1.9	82.7 ± 11.4	92.4 ± 8.1	90.0 ± 14.2	24.6 ± 3.2	>50/>50
2-FBU	0.9 ± 0.8	8.1 ± 3.5	0.2 ± 0.1	3.0 ± 1.6	2.2 ± 0.2	7.7 ± 0.3	>100	>100	64.8 ± 6.3	86.1 ± 24.0	40.9/>50
Sofosbuvir	0.6 ± 0.6	3.0 ± 2.5	1.0 ± 0.7	2.4 ± 1.5	ND	ND	>100	>100	85.6 ± 11.6	>100	>50/>50

^*a*^ Detection of YFV genomes in infected cell supernatant by qRT-PCR.

^*b*^ Determination of compound cytotoxicity by MTS assay.

^*c*^ Determination of compound mitochondrial toxicity by the 14-day mitochondrial toxicity assay.

^*d*^ CC_50_ = concentration displaying 50% cytotoxicity compared with untreated.

^*e*^ *EC* = 50% (EC_50_) or 90% (EC_90_*) effective antiviral concentration*.

^*f*^ PBM = human peripheral blood mononuclear cells. Vero = African green monkey kidney epithelial cells. Huh-7 = human hepatoma cells. MDM = primary human monocyte-derived macrophages. 3D spheroids = Huh-7 cells embedded in Matrigel.

^*g*^ IC_50_ MtDNA/nDNA = concentration displaying 50% inhibition of mitochondrial DNA compared with nuclear DNA in HepG2 cells. Results with 2′,3′-dideocytdine as a positive control for the mitochondrial toxicity assay demonstrated significant inhibition (. Xx%) at 10 µM. ND, not determined.

### Molecular modeling of DFA-DP binding to the YFV RdRp

**Fig 2:**
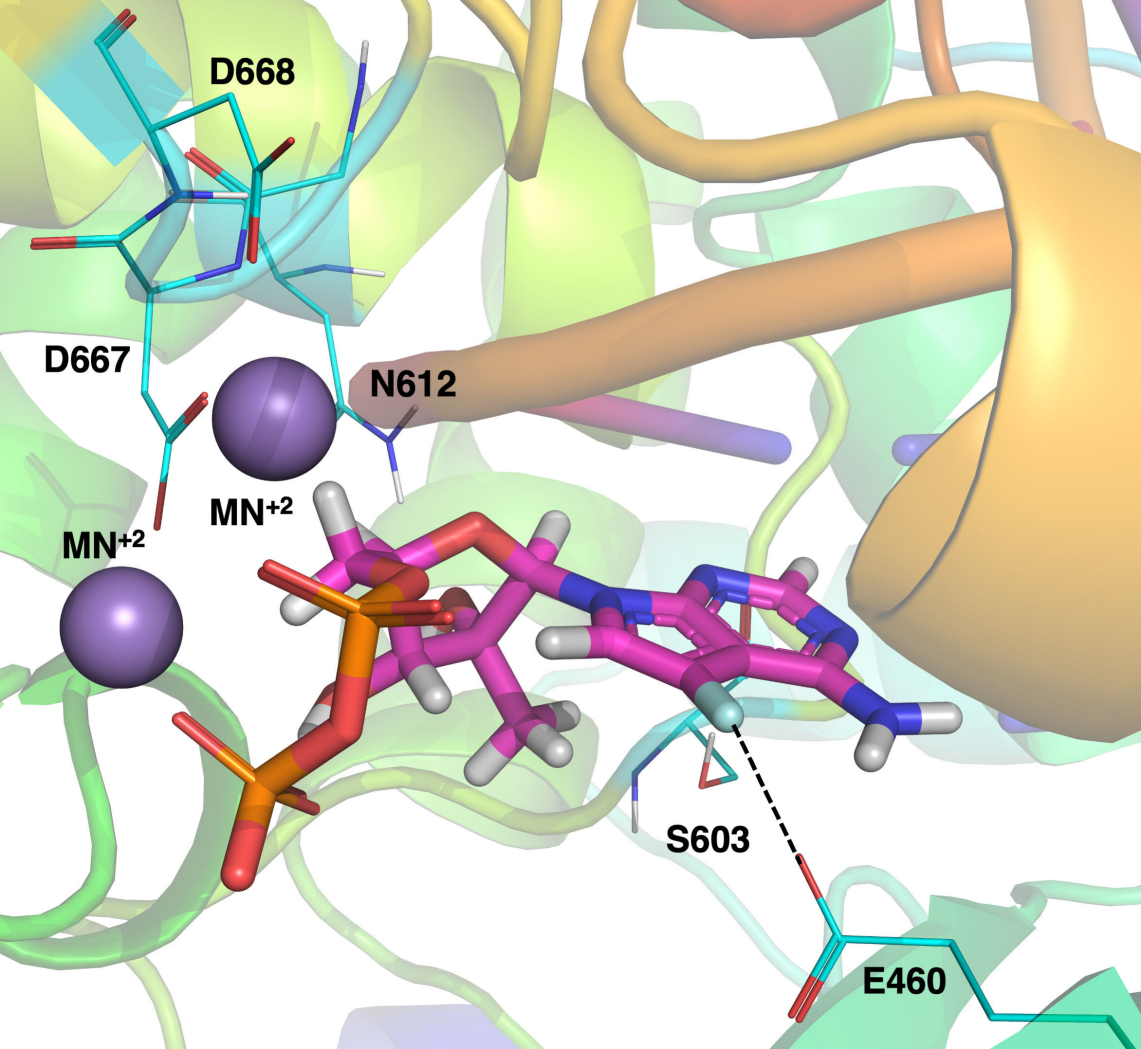
Molecular model predicting the binding of DFA-DP in the YFV RdRp catalytic pocket*.* Active site residues predicted to be involved in DFA-DP binding are highlighted. Shown residues: F atom of the DFA halogen bonds (dashed line) with E460 of the active site. Manganese co-factors (purple) bind residues D667 and N612 of the active site.

As a nucleoside analog, DFA-TP is expected to bind the active site of the viral RdRp to inhibit viral replication. To generate a predictive model of DFA binding, nucleoside conformation within the active site was transferred from the structurally homologous HCV RdRp previously crystallized using ADP in lieu of ATP (25). The nucleotide diphosphates exhibit enzymatically competent conformations consistent with the common RdRp mechanism (25). As such, molecular modeling was performed with ADP and DFA-DP to identify residues in the YFV RdRp critical for DFA’s activity. It is well-established that the 2′-Me group is a key modification leading to the antiflavivirus activity of these series of compounds, preventing new nucleotide entry into the RdRp (28). The relative positioning of the 2′-Me of DFA within the RdRp active site retains this positioning in our model. The fluorine on the base of DFA is predicted to halogen bond with E460 of the YFV RdRp (Fig. 2 dashed line). Additional aspartic acids D667 and D668 of the RdRp are predicted to bind manganese catalytic cofactors previously co-crystalized with the RdRp structure (24) (Fig. 2 purple spheres). Prime MM-GBSA dG (NS) values were calculated from Schrödinger. DFA-DP values were not different (−103.30 kcal/mol) from the natural substrate, ADP (−101.51 kcal/mol). Alignment of the YFV RdRp active site across 16 different clinical isolates and the vaccine strains 17D and 17DD showed 100% conservation of these residues. These residues are highly conserved across the flaviviruses, including WNV, DENV, ZIKV, JEV, and HCV (24).

### *In vitro* anti-YFV activity of DFA against clinical isolate DakH1279

**Fig 3:**
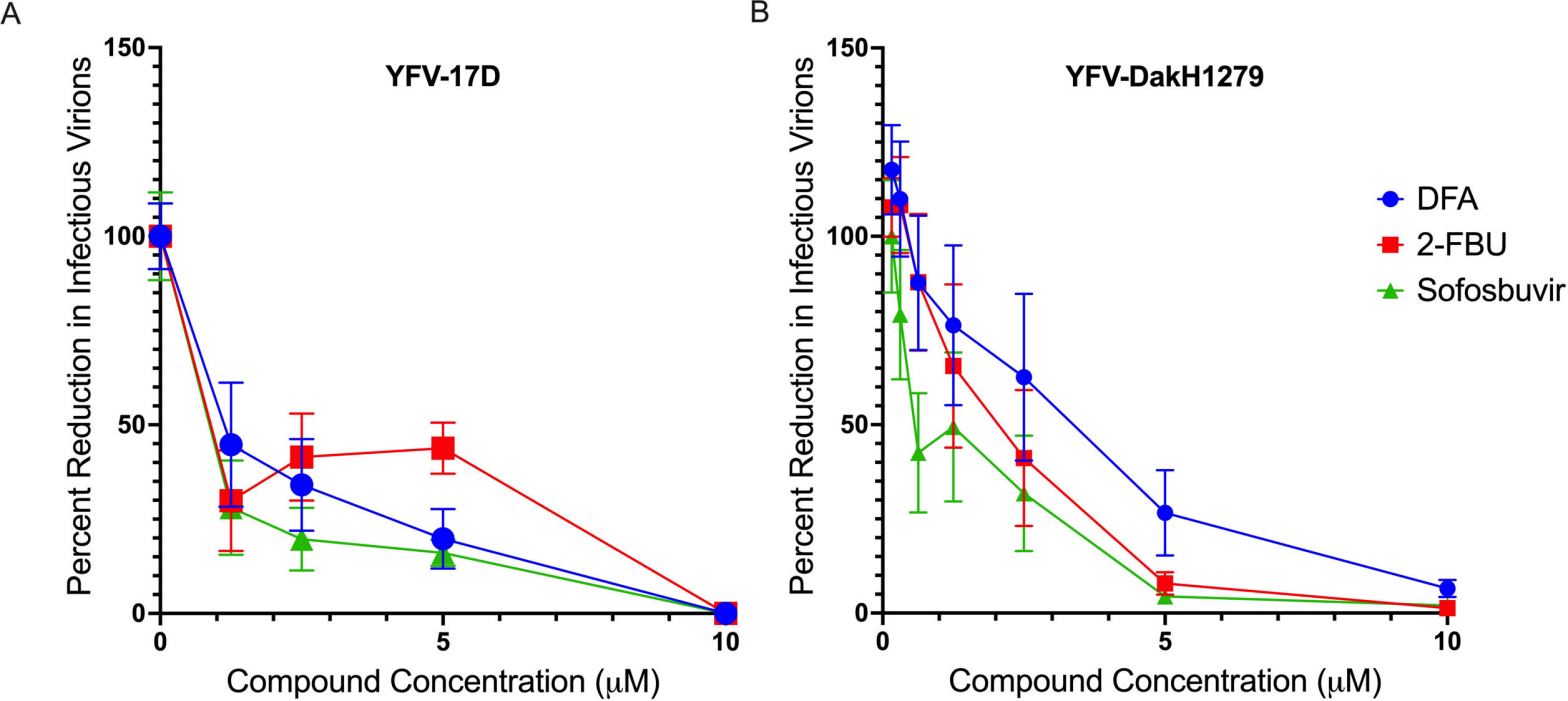
DFA reduces levels of infectious YFV yields in Huh-7 cells. (**A, B**) Huh-7 cells were infected with 260 FFU of YFV-17D (**A**) or 500 FFU YFV-Dak1279 (**B**) in a 96-well plate at 90% confluency. (**A**) After a 2 h incubation, the virus was removed, and cells were treated with twofold serially diluted compound (0–10 µM) in 1% methyl cellulose. After 3 days, cells were fixed, and foci were detected by ELISpot assay. (**B**) After a 2 h incubation, the virus was removed, and the cells were treated with twofold serially diluted compound (0–10 µM). After 2 days, infected cell supernatants were transferred to a new 96-well plate of Huh-7 cells to quantify infectious virus via foci-forming assay. (**A, B**) Foci were enumerated using an ImmunoSpot CTL reader, and EC50/EC90 values were calculated by linear regression on GraphPad Prism. *n* = 6. Shown mean ± standard error of the mean.

Given the high degree of homology of the RdRp across YFV strains (98.04% as determined by multiple sequence alignment from Brazilian epidemic strains (2016–2019) [10]), we investigated if DFA would retain its anti-YFV activity against a virulent clinical isolate. 2-FBU and sofosbuvir were also investigated, as they were not tested against clinical isolates previously. Compounds were evaluated *in vitro* for their ability to reduce yields of infectious virus against both YFV-17D and the highly lethal (92% mortality in rhesus macaques), viscerotropic, macaque-adapted clinical strain YFV-DakH1279 (29). In Huh-7 cells, compounds showed robust anti-YFV-17D (DFA: EC_50_ = 0.8 ± 0.4 µM, EC_90_ = 5.6 ± 2.6 µM; 2-FBU: EC_50_ = 1.2 ± 0.5 µM, EC_90_ = 8.6 ± 3.1 µM; sofosbuvir: EC_50_ = 0.3 ± 0.1 µM, EC_90_ = 1.2 ± 0.1 µM; Fig. 3A; Table 2) and anti-YFV-DakH1279 activity (DFA: EC_50_ = 2.4 ± 1.9 µM, EC_90_ = 5.0 ± 3.1 µM; 2-FBU: EC_50_ = 1.7 ± 1.1 µM, EC_90_ = 3.5 ± 2.3 µM; sofosbuvir: EC_50_ = 1.6 ± 1.3 µM, EC_90_ = 3.1 ± 2.4 µM; Fig. 3B; Table 2). 2-FBU had slightly higher EC_50_/EC_90_ values than both 2-DFA and sofosbuvir when tested against YFV-17D, and DFA had slightly higher EC_50_/EC_90_ values than both 2-FBU and sofosbuvir when tested against YFV-DakH1279. However, this difference was not statistically significant as determined by one-way ANOVA with Dunnett’s multiple-comparison test. Obtained values for infectious yields in FFU/mL revealed a 1.97 (98.9%), 4.23 (99.9%), and 4.43 (99.9%) log reduction at 10 µM for DFA, 2-FBU, and sofosbuvir, respectively (Table S1). With all compounds, there was a reduction in not only foci number but also foci size with increased compound concentration (Fig. 1), demonstrating that the compounds limited both replication and spread within the culture. As the FFU measure used to quantify the total virus yield under each condition only accounts for the presence/absence of infectious foci, the obtained values as FFU/mL (Fig. S1) are likely an underestimation of potency.

**TABLE 2:** Antiviral activity of YFV 17d and DakH1279 strains as determined by reduction in infectious virus

	Anti-YFV-17D		Anti-YFV-DakH1279	
Compound	EC_50_ (μM)	EC_90_ (μM)	EC_50_ (μM)	EC_90_ (μM)
DFA	0.8 ± 0.4	5.6 ± 2.6	2.4 ± 1.9	5.0 ± 3.1
2-FBU	1.2 ± 0.5	8.6 ± 3.1	1.7 ± 1.1	3.5 ± 2.3
Sofosbuvir	0.3 ± 0.1	1.2 ± 0.1	1.6 ± 1.3	3.1 ± 2.4

### *In vivo* activity of DFA against YFV-17DD in A129 mice

**Fig 4:**
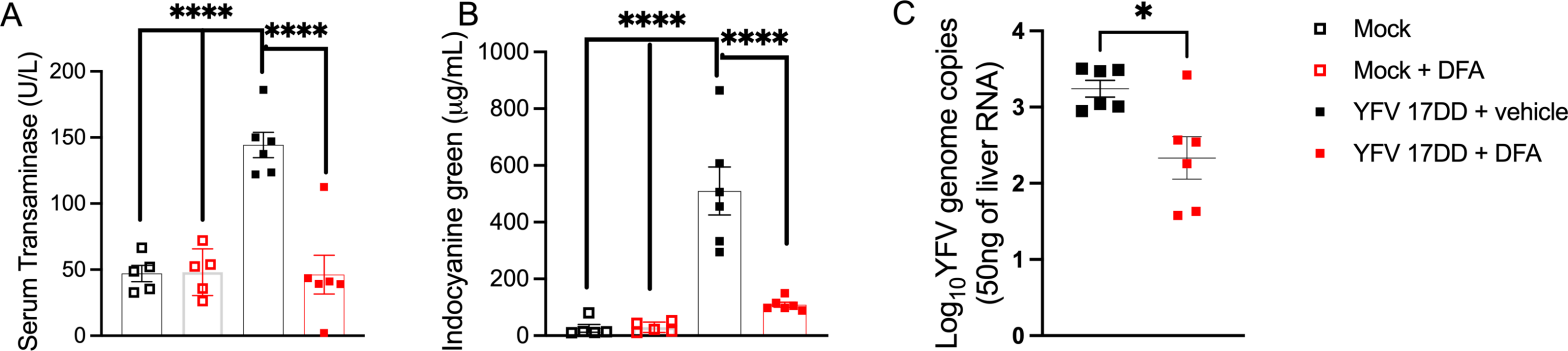
DFA’s anti-YFV-17DD activity in A129 mice*.* Equal numbers of male and female A129 mice aged 8–10 weeks (six mice in total) were infected i.p. with 10^6^ PFU of YFV-17DD in the presence or absence of 10 mg/kg of DFA, injected i.v. 1 h before infection, and every 24 h for 3 days thereafter. Control mock and mock + DFA groups had five mice (three males, two females; *n* = 5 per group). **A**) Liver alanine aminotransferase (ALT) levels measured by colorimetric assay from murine sera, expressed as U/mL. Statistical differences among groups were assayed by one-way ANOVA plus Dunnett’s multiple-comparison test. **B**) Indocyanine green (ICG) clearance on day 3 before euthanasia. Mice were administered 20 mg/kg ICG 20 m before euthanasia. After euthanasia, blood was collected from the vena cava; serum was isolated; and ICG levels were quantified by spectrophotometry. **A, B**) Statistical differences between groups were determined by one-way ANOVA plus Dunnett’s multiple-comparison test. Shown mean ± standard error of the mean (SEM); ****P > 0.0001. **C**) qRT-PCR to assess viral load in the murine liver tissue 3 dpi, following euthanasia. Statistical significance between groups assessed by two-tailed unpaired Student *t*-test. Shown mean ± SEM; *P = 0.0128.

The data herein support DFA as a potent inhibitor of YFV; however, as was demonstrated with sofosbuvir, *in vitro* data do not necessarily translate to *in vivo* efficacy (9). To evaluate the *in vivo* potential of DFA, a type I interferon receptor-deficient murine model (A129) infected with YFV-17DD was used (30, 31). To provide a comparative measure with previously demonstrated 2-FBU’s anti-YFV-17DD activity in A129 mice, the same experimental design was employed (10). In brief, equal numbers of male and female mice were infected i.v. in the tail vein with YFV-17DD (vaccine strain; 10^6^ PFU), and then treated with 10 mg/kg i.p. of DFA at 1 h pi and every 24 h for 3 days. Control groups received YFV + saline or were mock-infected (C6/36 cell supernatant only). Over 3 days, there were no significant changes in body weight (Fig. S2A) or total white blood cell numbers (Fig. S2B) across all groups, typical of a mild YFV-17DD infection in A129 mice (30, 31). Infected mice exhibited YFV-17DD-associated liver pathology. Alanine aminotransferase (ALT) enzyme levels were measured as an indication of liver damage. ICG clearance rate from systemic circulation was measured to evaluate liver dysfunction. Treatment with DFA significantly reduced ALT levels (Fig. 4A; *P* < 0.0001) and improved ICG clearance (Fig. 4B; *P* < 0.0001). Importantly, DFA administration significantly reduced YFV-17DD replication in the livers of infected mice (Fig. 4C; *P* = 0.013). Levels of inflammatory cell infiltration (infiltrate score ~1) were roughly equivalent across infected groups, regardless of treatment (Fig. S3A). Histopathological analysis revealed reduced liver pathology (cellular infiltration, hepatocyte swelling, necrosis, hemorrhage, and degeneration) in infected mice receiving DFA compared to those infected but not receiving treatment, though this reduction was insignificant (Fig. S2B through F). This is often seen with the mild and self-limiting infection produced by vaccine strain (9, 30, 31). DFA’s demonstrated anti-YFV-17DD activity in the A129 mouse model was similar to that as previously shown for 2-FBU (0.7 log, ~80% reduction in YFV genome copies present in the liver tissue) (10).

### *In vivo* activity of DFA against a YFV clinical isolate in AG129 mice

**Fig 5:**
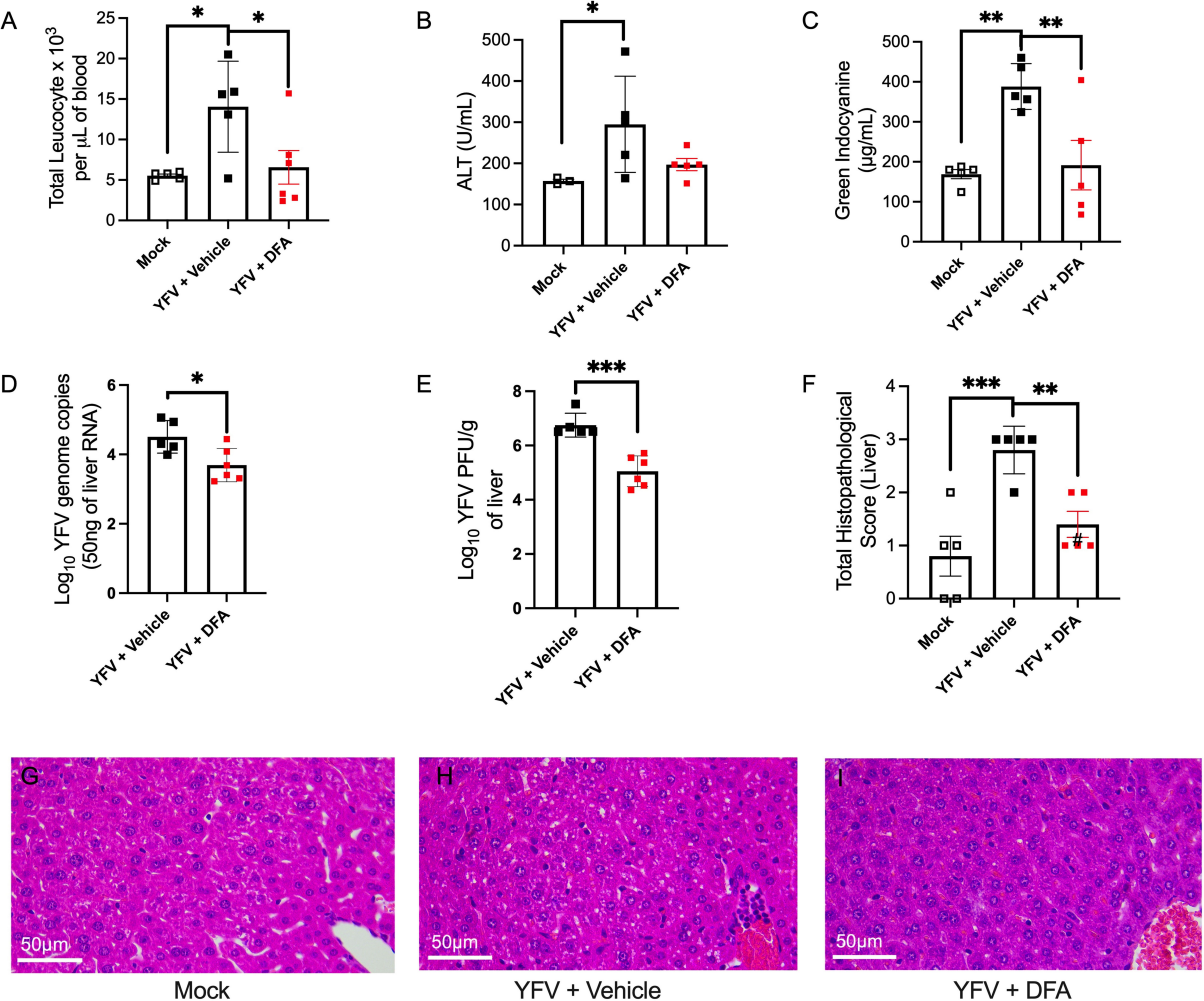
DFA’s anti-wt-YFV activity in AG129 mice. Equal numbers of male and female AG129 mice (*n* = 6) aged 8–9 weeks were infected i.p. with 10^3^ PFU of clinical YFV isolate WT306. At 1 hpi and every 24 h thereafter, mice were administered i.v. 10 mg/kg of DFA or saline (vehicle control) for 5 days after which mice were sacrificed, and blood and tissues were collected and analyzed. An uninfected mock group with three males and two female mice was used as a negative control (*n* = 5). (**A**) Quantification of the total circulating leukocytes. Leukocytes were isolated from the murine blood collected from the vena cava and counted. Mean counts from the YFV-infected group were compared with mean counts from mock or YFV + DFA groups by one-way ANOVA plus Šídák’s multiple-comparison test. Shown, mean ± standard error of the mean (SEM); *P = 0.019 (mock vs YFV + vehicle), *P = 0.03 (YFV + vehicle vs YFV + DFA). (**B**) Liver alanine aminotransferase (ALT) levels measured by colorimetric assay from murine sera, expressed as U/mL. Statistical differences were assayed by one-way ANOVA plus Kruskal–Wallis multiple-comparison test. Shown mean ± SEM; *P = 0.03 (mock vs YFV + vehicle). (**C**) Indocyanine green (ICG) clearance on day 5 before euthanasia. Mice were administered 20 mg/kg ICG 20 min before euthanasia. After euthanasia, blood was collected from the vena cava; serum was isolated; and ICG levels were quantified by spectrophotometry. Statistical differences between groups were determined by one-way ANOVA plus Dunnett’s multiple-comparison test. Shown mean ± SEM; **P = 0.036 (mock vs YFV + vehicle), **P = 0.0075 (YFV + vehicle vs YFV + DFA). (**D**) qRT-PCR to assess viral load in liver tissue. Statistical significance between groups assessed by two-tailed unpaired Student’s *t*-test. Shown mean ± SEM; *P = 0.02. (**E**) Quantification of infectious viral particles in liver tissues via plaque assay on Vero cells, expressed as PFU/mL. Statistical significance between groups assessed by two-tailed unpaired Student’s *t*-test. Shown mean ± SEM; ***P = 0.0004. (**F**) Histological grading of inflammatory infiltrates in murine livers. Statistical differences between groups were determined by one-way ANOVA plus Dunnett’s multiple-comparison test. Shown mean ± SEM; ***P = 0.0006 (mock vs YFV + vehicle), **P = 0.0081 (YFV + vehicle vs YFV + DFA). G–I) H&E-stained liver slice micrographs of (**G**) mock, (**H**) YFV + vehicle, and (**I**) YFV + DFA. Scale bar: 50 µM.

The promising *in vitro* and *in vivo* activity of DFA obtained with the clinical DakH1279 YFV strain and YFV-17DD supported further *in vivo* evaluation with a wt-YFV clinical isolate. The type I and II interferon receptor-deficient wt-flavivirus mouse model (AG129 [31]) was used to assess DFA’s anti-wt-YFV activity. Infections were carried out as described above with the A129 mice (3.4), but infection and DFA treatment were maintained for 5 instead of 3 days. All wt-YFV infected mice showed a significant increase in clinical disease scores, while DFA treatment provided a modest but significant decrease in clinical severity by day 5 (*P* = 0.0023; Fig. S1A). wt-YFV infection induced significant leukocytosis (*P* = 0.019 compared to mock; Fig. 5A), while DFA treatment significantly reduced not only leukocytosis (*P* = 0.034; Fig. 5A) but also the total portion of granulocytes in the blood (*P* = 0.049; Fig. S1) compared with mice not receiving DFA treatment (Fig. S4B). DFA also protected the liver. ALT levels were significantly increased in wt-YFV-infected mice (*P* = 0.032; Fig. 5B) and markedly reduced in infected mice receiving DFA treatment. While the reduction in ALT levels resulting from DFA treatment was not statistically significant (*P* = 0.06; Fig. 5B), this owed to an outlier within the data set. As another measure of the liver function, the ICG clearance was assessed. As expected, wt-YFV infection significantly reduced the ICG clearance (*P* = 0.0035; Fig. 5C), while DFA treatment significantly improved the ICG clearance (*P* = 0.0081; Fig. 5C). In the liver itself, DFA significantly reduced virus replication, as indicated by both a reduction in total genome copy numbers (*P* = 0.02; Fig. 5D) and a reduction in infectious virions (*P* = 0.0004; Fig. 5E). Histological analysis showed significant immune cell infiltration and steatosis (*P* = 0.0006; Fig. 5F and H) in wt-YFV infected mice, while DFA significantly reduced YFV-mediated liver pathology (*P* = 0.0081; Fig. 5F and I). While variability was noted owing to outliers, DFA administered daily at 10 mg/kg provided significant protection to AG129 mice, reducing wt-YFV replication and YFV-associated disease and pathology.

## DISCUSSION

Nucleoside analogs are a highly effective class of antiviral agents that mimic natural nucleosides and, in their 5′-triphosphate form, can selectively terminate viral DNA or RNA synthesis. Previously, we reported on a panel of 21 in-house synthesized modified nucleoside analogs as anti-YFV agents (8). 2-FBU and DFA were identified, among others, to have a sub-micromolar activity against YFV and limited cytotoxicity *in vitro* (8). DFA was particularly attractive, as it exhibits pan-anti-flavivirus potential with favorable pharmacokinetics in male rats (12–14). Originally developed as an anti-HCV drug (14), DFA is active against other flaviviruses (12, 13) and, herein, we extend this activity profile to YFV for potential preclinical development.

Several attempts have been made to repurpose or design new nucleoside analogs to treat YFV. Galidesivir, an adenosine analog approved in Japan for the treatment of influenza, was active against YFV *in vitro* (32) and included in a prematurely terminated clinical trial in 2019 (NCT03891420 [33]). Sofosbuvir has anti-YFV activity *in vitro*. However, it was only effective in mice when given prophylactically (9). Interestingly, isosteric replacement of the 2′-methyl on the sugar moiety of sofosbuvir with a bromine atom (2-FBU) (8) enhanced potency and conferred protection to YFV-17DD-infected A129 mice (10). Given sofosbuvir and 2-FBU’s anti-YFV activity, this study used them as controls for *in vitro* evaluations. DFA is a 2′-C-methyl nucleoside analog with a 7-deaza-7-fluoro purine base. While the corresponding 2′-C-methyl-adenosine analog was shown to have potent anti-HCV activity, replacement of the natural adenine base in that compound with 7-deaza-7-fluoro adenine enhanced its bioavailability and stability by conferring resistance to enzymatic conversion by phosphorylases and deaminases in male rats, making DFA a candidate for oral dosing (14).

Compounds were initially evaluated *in vitro* in relevant 2D and 3D human hepatoma models and primary human macrophages. Macrophages are a critical cell type in acute infection, acting as an early recipient and reservoir for the virus following initial infection (4). Macrophages also transport viruses through the lymph to target liver organs (4). Over 50% of lethal YFV cases involve liver damage/destruction and jaundice from which the virus derives its name (2–5). As expected, DFA and 2-FBU were more potent in 2D hepatoma cultures compared with our 3D Matrigel model (DFA: EC_50_ = 0.9 vs. 5.5 µM; 2-FBU: EC_50_ = 0.9 vs 2.2 µM), though potency was well within a therapeutic window. In primary human macrophages, compounds were consistently more active than hepatomas, particularly reflected in the EC_90_ values (DFA: EC_90_ = 1.0 vs. 3.4 µM; 2-FBU: EC_90_ = 3.0 vs 8.1 µM). In all cases, results for 2-FBU were consistent with our previous reports (10). Given the role of macrophages in promoting systemic dissemination of YFV during infection, it is prudent to develop antiviral compounds that can limit viral replication/spread within this population. To date, considerations of macrophage targeting in anti-YFV drug development are widely overlooked. To our knowledge, this and our previous work on 2-FBU (10) are the first accounts of nucleoside analogs with anti-YFV activity in macrophages. Activity data derived from 3D cultures are generally more predictive of *in vivo* efficacy owing to enhanced biological relevance over traditional 2D immortalized monolayer cell cultures. As such, we validated 2D activity data in an in vivo-like 3D and/or primary system before studying the nucleoside analogs in animal models.

The A129 murine model was initially used to evaluate DFA’s *in vivo* efficacy against YFV-17DD. Mice were evaluated at 3 dpi, receiving 10 mg/kg of DFA initially at 1 hpi, followed by daily dosing. Dosage was determined by previous studies in ZIKV-infected A129 mice receiving DFA treatment (13). As expected, YFV-17DD produced a mild infection in A129 mice with no notable adverse effects. Administration of DFA had no notable impact on the overall health status. Importantly, DFA was protected from YFV-17DD-induced liver impairment, significantly reducing virus burden and markers of liver damage in YFV-17DD-infected mice. In this system, we could not determine protection from liver pathologies, such as steatosis, necrosis, or apoptosis, as these were not readily present in infected mice, regardless of treatment. While these gross pathological indicators are often present in this model, the mild nature of infection tends toward variability. DFA’s protection against liver damage and ability to reduce viral burden in A129 mice was comparable to that as previously shown for 2-FBU in the same model utilizing the same dosing strategy (10). While sofosbuvir has repeatedly shown potent *in vitro* antiviral activity (8–11), sofosbuvir failed to provide protection and reduce anti-YFV activity in the A129 mouse model (9). These data provided apt justification for continued studies of DFA’s and 2-FBU’s efficacy against clinical strains. Within the scope of this study, we chose to further evaluate DFA’s anti-YFV activity *in vivo* against a virulent clinical isolate of YFV.

Our *in vitro* and *in vivo* data with vaccine strain, coupled with molecular modeling predicting DFA binding to the highly conserved YFV RdRp binding pocket, suggested potential activity against clinical strains. Indeed, DFA and 2-FBU significantly reduced the replication of YFV-DakH1279 in human hepatoma cultures. To assess *in vivo* efficacy of DFA against a clinical YFV strain, the AG129 mouse model was employed. In this model, wild-type YFV infection mirrors human and non-human primate (NHP) infection without animal-specific virus adaptation, thereby enhancing relevance (31). Overall, by day 5, all infected mice showed evidence of clinical disease, though there was a slight but significant decrease in disease phenotype in mice receiving DFA treatment. More telling was the significant protection from YFV-associated liver disease (dysfunction, inflammation, and steatosis) concurrent with a significant reduction in viral load using the same 10 mg/kg daily dosing strategy as was used with YFV-17DD. A limitation to this study was the employment of a single dosing strategy in the murine model (i.e., compound administered at a single dose 1 h post-infusion and every 24 h thereafter). However, the data collected from this work provide valuable insight into DFA’s potential as an anti-YFV agent and serve as the groundwork to justify pursuing DFA in advanced studies evaluating route of drug administration (oral vs. i.p. injection) and timing of intervention post-infection. Together with *in vitro* anti-YFV-DakH1279 results, these data support further investigation of these parameters in a murine model and in an NHP model.

Dubankova and Boura (24) resolved the crystal structure of YFV RdRp and reported high homology (>65%) with other vector-borne flaviviruses (24). Given this, a drug targeting the viral polymerase could be an efficacious pan-flavivirus therapeutic option (24). Several 2′-Me modified nucleoside analogs have been shown to display anti-flavivirus activity by inhibiting chain elongation after binding to the viral RdRp (as reviewed by [34]). Our *in silico* model shows that, in the case of DFA, the 2′-Me positioning remains similar to that of these compounds, but that the 7-fluorine substitution affords additional halogen bond formation between the fluorine atom and residue E460 of the YFV RdRp active site. In contrast, the bromine atom at 2′-C of compound 2-FBU’s base is predicted to form a strong halogen bond with residue D668 of the YFV active site (10). Further developing both compounds is prudent, as RNA viruses are notorious for acquiring mutations. Indeed, YFV exists as a viral quasispecies with many closely related mutants grouped (35). Rational drug design implies that mutations conferring resistance may i) already be present in the population or ii) develop under selective pressure. Following this, treatment can become the fuel for generating more virulent and harder-to-treat virus strains. Thus, the development of two structurally different nucleoside analogs, such as 2-FBU and DFA, could be beneficial, as it is expected that they would have a different mutation profile and could potentially be used in combination therapy approaches with different classes of antivirals, such as neutralizing monoclonal antibodies (29), to combat the emergence of resistant mutants. Drug-resistant virus selection and biochemical studies to address this are ongoing.

### Conclusions

In summary, DFA significantly reduced signs of liver damage and viral burden in mice infected with either a vaccine strain or a clinical isolate modeling severe human disease. Molecular modeling predicted DFA-5′-diphosphate binding to the YFV RNA-dependent RNA polymerase using active site residues highly conserved between clinical YFV strains and across flaviviruses. In addition, we validated our previous findings on the anti-YFV activity of 2-FBU against YFV-17D (10) and reported herein the antiviral activity against the virulent YFV-DakH1279 strain. These data support the further preclinical development of DFA and 2-FBU as anti-YFV agents and of DFA as a pan anti-flavivirus agent based on its broad-spectrum activity against flaviviruses such as HCV, WNV, DENV, ZIKV, and JEV.

This work contributes to the larger goal of discovering safe and efficacious nucleoside analog therapies for the treatment of YFV. DFA also has a broad-spectrum activity against flaviviruses, such as HCV, WNV, DENV, ZIKV, and JEV, making it an attractive compound for advanced preclinical work. The successful discovery and development of potent YFV, such as DFA, have a far-reaching impact as an adjunct to vaccination and an opportunity to curb future epidemics resulting from the migration of YFV-harboring mosquitoes into largely unvaccinated urban areas and/or regions with waning immunity.

## SUPPLEMENTAL MATERIAL

Supplemental material aac.01889-24-s0001.docx Table S1; Fig. S1 to S4.

ASM does not own the copyrights to Supplemental Material that may be linked to, or accessed through, an article. The authors have granted ASM a non-exclusive, world-wide license to publish the Supplemental Material files. Please contact the corresponding author directly for reuse.
