# Supplementary material for: Combating yellow fever virus with 7-deaza-7-fluoro-2′-C-methyladenosine
Source: Antimicrob Agents Chemother. 2025 Apr 14;69(5):e01889-24. doi: 10.1128/aac.01889-24 (PMC12057363; doi:10.1128/aac.01889-24)
Supplement: Supplemental material — Table S1; Fig. S1 to S4. [file aac.01889-24-s0001.docx]

| Concentration (μM) | YFV-17D | | | YFV-DakH1279 | | |
| --- | --- | --- | --- | --- | --- | --- |
|  | DFA  (FFU/mL) | 2-FBU  (FFU/mL) | Sofosbuvir  (FFU/mL) | DFA  (FFU/mL) | 2-FBU  (FFU/mL) | Sofosbuvir  (FFU/mL) |
| 0 | 1.8x10^5^ ±  3.4x10^4^ | 1.8x10^5^ ± 3.4x10^4^ | 1.8x10^5^ ± 3.4x10^4^ | 4.8x10^4^ ± 2.0x10^4^ | 4.8x10^4^ ± 2.0x10^4^ | 4.8x10^4^ ± 2.0x10^4^ |
| 1.25 | 1.2x10^5^ ±  3.5x10^4^ | 1.2x10^5^ ± 3.5x10^4^ | 9.8x10^4^ ± 3.4x10^4^ | 5.5x10^4^ ± 9.9x10^3^ | 4.0x10^4^ ± 6.1x10^3^ | 2.7x10^4^ ± 1.1x10^4^ |
| 2.5 | 1.0x10^5^ ±  2.8x10^4^ | 1.1x10^5^ ± 3.9x10^4^ | 7.6x10^4^ ± 4.9x10^4^ | 4.7x10^4^ ± 4.9x10^3^ | 2.4x10^4^ ± 9.8x10^3^ | 1.4x10^4^ ± 1.2x10^4^ |
| 5 | 4.9x10^4^ ±  2.9x10^4^ | 8.9x10^4^ ± 2.8x10^4^ | 2.0x10^4^ ± 2.2x10^4^ | 2.4x10^4^ ± 8.0x10^3^ | 3.3x10^3^ ± 2.4x10^3^ | 2.2x10^3^ ± 1.2x10^3^ |
| 10 | 0 | 0 | 0 | 5.1x10^3^ ± 1.9x10^3^ | 2.8x10^2^ ± 1.7x10^2^ | 1.8x10^2^ ± 1.2x10^2^ |

Supplemental Table 1: Avergae infectious YFV yields in FFU/mL in the presence of increasing concentrations of compound.


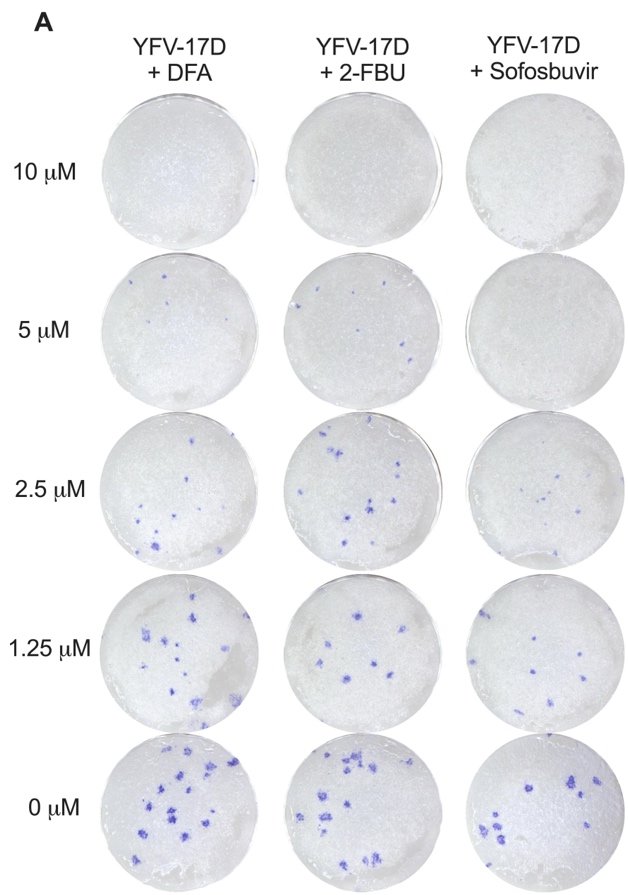

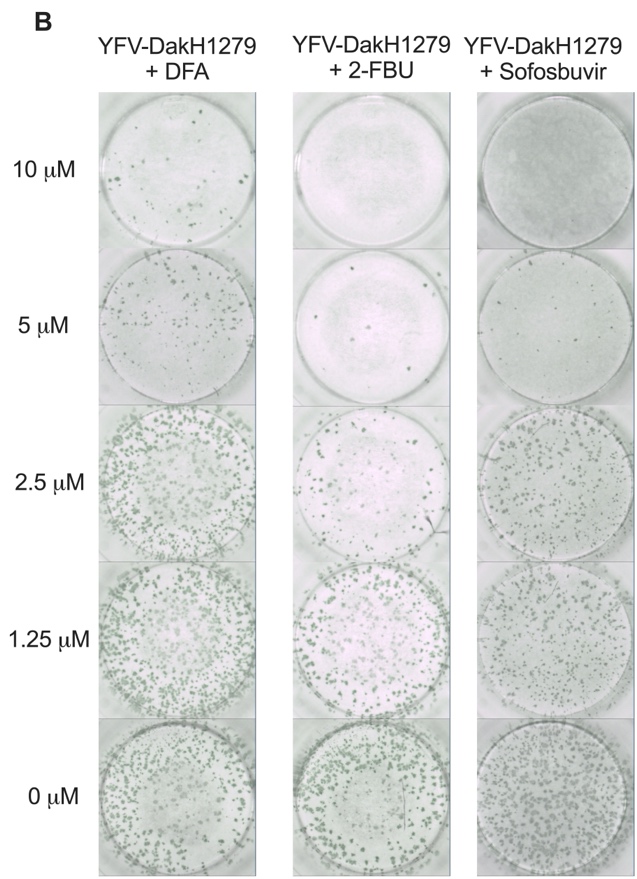


Supplemental Figure 1: Antiviral ELIspot assay to assess compound dose-dependent anti-YFV-17D (A) and anti-YFV-DaKH1279 (B) activity in Huh-7 cells. Huh-7 cells were infected with YFV-17D (260 FFU/mL) or YFV-DaKH1279 (500 FFU/mL) for 3 (YFV-17D) or 2 (DakH1279) days in the presence of 2-fold serially diluted compound (0 – 10µM), then fixed with 4% PFA and virus antigen detected by ELIspot and imaged and quantified on a CTL reader. Following Immunospot quantification, values were convereted to infectious virus yields as FFU/mL. Shown, representative micrographs from YFV-17D (A) and YFV-DakH1279 (B) antiviral ELIspot assays.


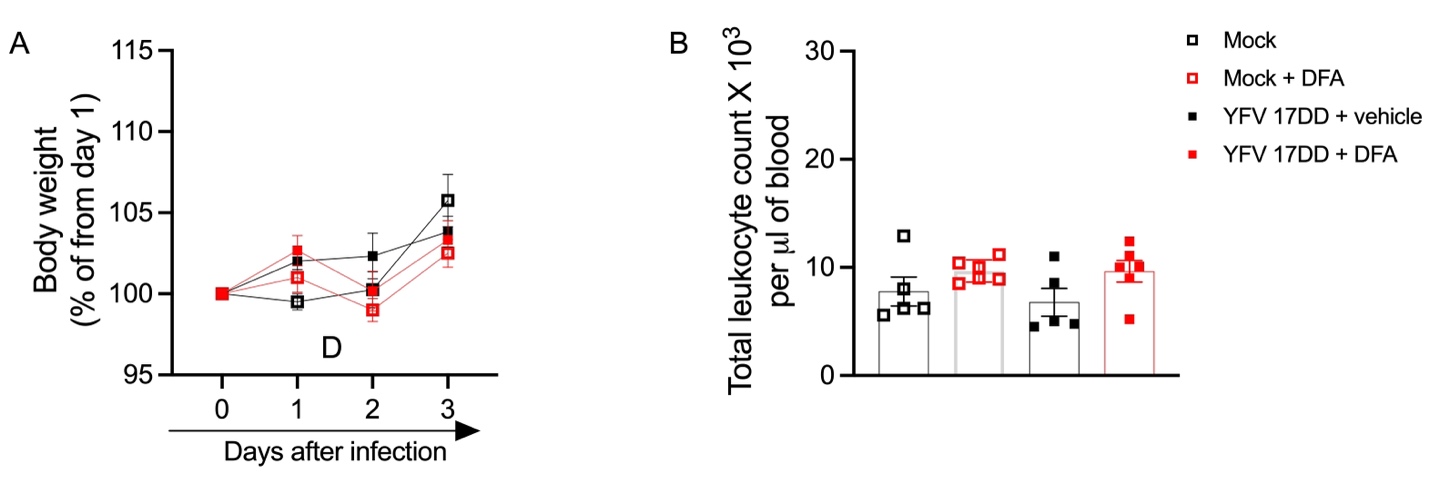


Supplemental Figure 2: Daily body weight monitoring and final (day 3) leukocyte counts of YFV-17DD infected mice with or without DFA treatment. Equal numbers of male and female A129 mice aged 8 – 10 weeks (6 mice total) were infected ip with 10^6^ PFU of YFV-17DD in the presence or absence of 10 mg/kg of DFA, injected iv 1 hr before infection and every 24 hr for 3 days thereafter. A) Body weight changes post-infection was monitored daily and analyzed as percentage of body weight from day 0, before infection. Changes in body weight were assessed by one-way ANOVA plus Šídák’s multiple comparisons test. Shown mean ± SEM. B) Total circulating leukocyte counts from blood isolated from the vena cava at the time of euthanasia. Data from each group was compared to either mock or mock + DFA using one-way ANOVA plus Tukey’s multiple comparisons test. Shown mean ± SEM.


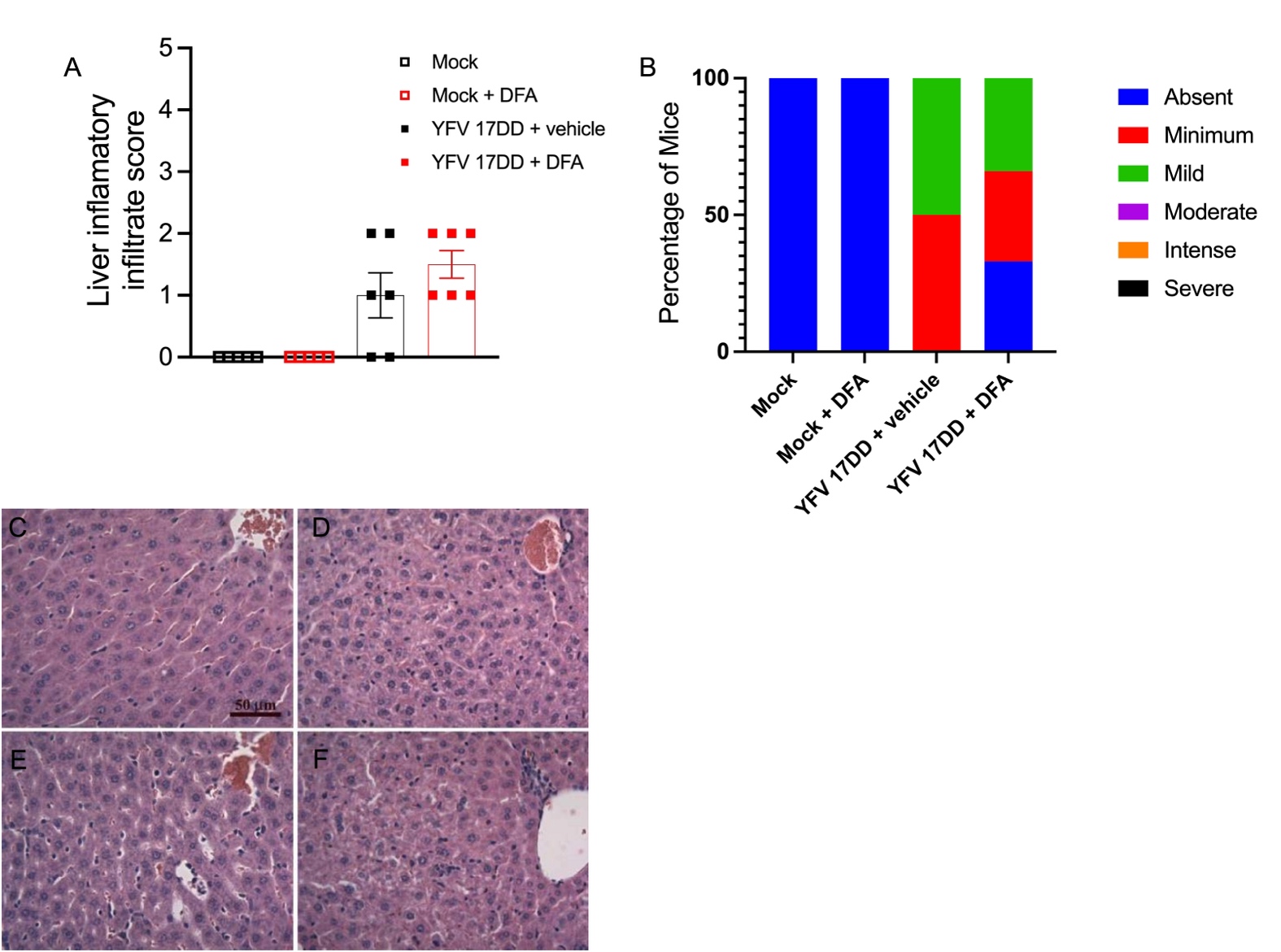


Supplemental Figure 3. Assessment of liver immune cell infiltration of YFV-17DD infected mice with or without DFA treatment. Equal numbers of male and female A129 mice aged 8-10 weeks (6 mice total) were infected i.p. with 10^6^ PFU of YFV-17DD in the presence or absence of 10 mg/kg of DFA, injected iv 1 hr before infection and every 24 hr for 3 days thereafter. After euthanasia, livers were collected and processed for immunohistochemistry with H&E staining. A) Quantification of immune cell infiltration in murine liver sections. Shown mean ± SEM. B) Histopathological scoring of liver sections, 2 per animal. A four-point scoring system was used (0 = absent, 1 = slight, 2 = moderate, 3 = marked, 4 = severe). C – F) Representative micrographs of H&E-stained liver sections. C) mock, D) mock + DFA, E) YFV 17DD + vehicle, F) YFV 17DD + DFA. Scale bar: *5*0 µM.


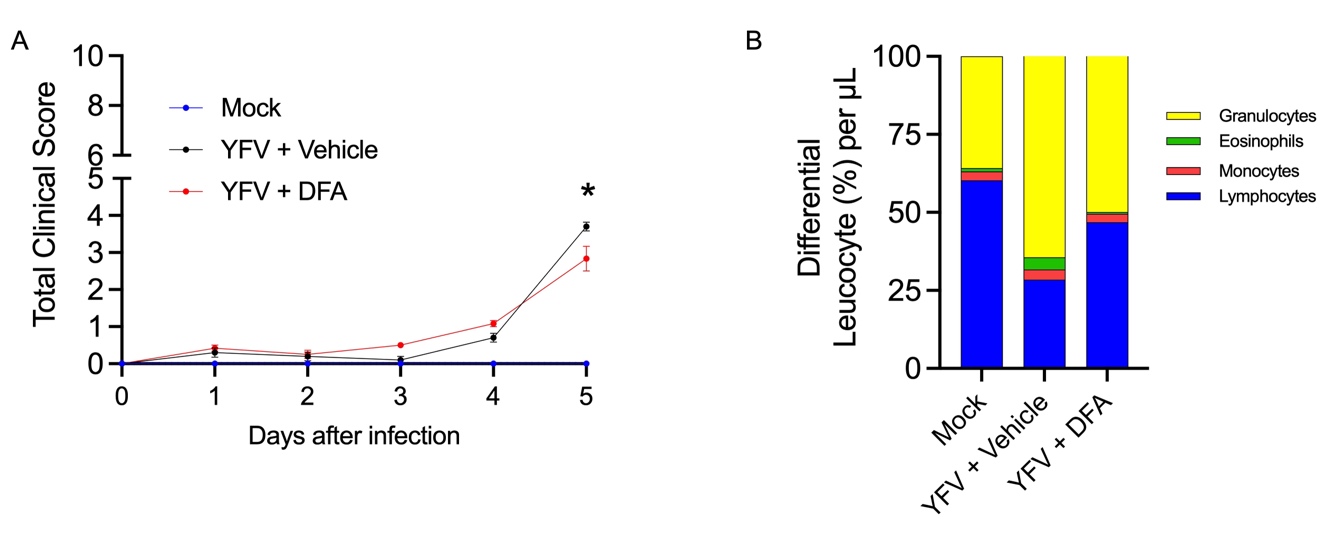


Supplemental Figure 4: Daily body weight monitoring and final (day 5) distribution of white blood cell phenotypes of wt-YFV infected mice with or without DFA treatment. Equal numbers of male and female AG129 mice aged 8 – 9 weeks (6 mice total per infected group; 5 mice in mock group) were infected i.p. with 10^3^ PFU of wt-YFV in the presence or absence of 10 mg/kg of DFA, injected iv 1 hr before infection and every 24 hr for 5 days thereafter. A) Body weight changes post-infection were monitored daily and analyzed as percentage of body weight from day 0, prior to infection. Changes in body weight were assessed by fitting a mixed model plus Tukey’s multiple comparisons test. Shown mean ± SEM. B) Total circulating leukocyte were differentiated into phenotypes of granulocytes, eosinophils, monocytes, and lymphocytes. Shown is a sub-population percentage of total leukocytes.
